# Exploring associations between older adults’ demographic characteristics and their perceptions of self‐care actions for communicating with healthcare professionals in southern United States

**DOI:** 10.1002/nop2.315

**Published:** 2019-06-17

**Authors:** Huey‐Ming Tzeng, Udoka Okpalauwaekwe, Cindy Feng, Sandra Lynn Jansen, Anne Barker, Chang‐Yi Yin

**Affiliations:** ^1^ School of Nursing The University of Texas Medical Branch Galveston Texas; ^2^ College of Medicine University of Saskatchewan Saskatoon Saskatchewan Canada; ^3^ School of Public Health University of Saskatchewan Saskatoon Saskatchewan Canada; ^4^ College of Nursing University of Saskatchewan Saskatoon Saskatchewan Canada; ^5^ Whitson‐Hester School of Nursing Tennessee Technological University Cookeville Tennessee; ^6^ Taiwan History Research Foundation Taipei Taiwan

**Keywords:** communicating with healthcare professionals, patient engagement, patient involvement, patient‐centred care, self‐care

## Abstract

**Aims:**

This study examined associations between older adults’ demographic factors and their perceived importance of, desire to and ability to perform seven self‐care behaviours for communicating with healthcare professionals.

**Design:**

This cross‐sectional survey study analysed subset data of 123 older adults 65 years and older, living in southern United States.

**Methods:**

The *Patient Action Inventory for Self‐Care* (57 items, grouped into 11 categories) was used to collect self‐reported self‐care data. Demographic characteristics were also collected. Descriptive statistics and logistic regression analyses were used to tests for relationships between the variables relevant to the research objective.

**Results:**

Regression findings showed that separated older adults felt less able to share ideas about their healthcare experiences compared to married older adults. Male older adults reported less desire to list issues to discuss and less desire to share ideas about their care experience with their healthcare professionals compared to their female counterparts.

## 1 INTRODUCTION

Effective communication between patients and healthcare professionals (i.e., nurses, physicians, or other allied healthcare staff) is of important concern as it facilitates patient‐centred care delivery and promotes better quality health services (Hashim, 2017; Ozaras & Abaan, 2016; Tzeng, Okpalauwaekwe, & Yin, 2019). With nursing staff particularly, it has been shown empirically that satisfactory patient outcomes are associated with, and significantly reliant on the quality of patient communication with their healthcare professionals (Ausserhofer et al., 2013; Cené, Roter, Carson, Miller, & Cooper, 2009; Ozaras & Abaan, 2016; Pehrson et al., 2016; Wittenberg‐Lyles, Goldsmith, & Ferrell, 2013).

A recent study conducted by Tzeng, Pierson, Kang, Barker, and Yin (2019) found that community‐dwelling adults in the United States, who valued the importance of itemizing health‐related issues to discuss with their care providers, were less likely to have used emergency room services in the past 3 months. Additionally, adults who indicated that they were able to share their physical symptoms with their healthcare professionals were also less likely to have used emergency room services in the past three months (Tzeng, Pierson, et al., 2019). Similarly, a recent qualitative study exploring suggestions from older adults to promote patient‐engaged health delivery in western Canada revealed older adults’ desire to have a conducive clinical environment that fosters effective communication with their healthcare providers (Tzeng, Okpalauwaekwe, et al., 2019). As far as we know, there are no current studies that investigate the influences and contributions of patient demographic characteristics to these perceived perceptions of the self‐care actions related to communicating with their healthcare professionals in the United Stated and other countries.

### 1.1 Objectives and design of this study

In order to develop patient‐centred health policies and improve health practices for the older adult population in general, it will require an understanding of older adults’ perceptions of self‐care actions that are needed for them to communicate with healthcare professionals, as well as the associations between their perceptions and demographic characteristics. Therefore, the purpose of this study was to explore the relationships between community‐dwelling older adults’ demographic characteristics and their perceptions of self‐care actions for communicating with healthcare professionals in the southern parts of the United States. The main research question of this study is *Are older adults’ demographic characteristics associated with their perceived importance levels of self‐care actions for communicating with healthcare professionals, as well as their desire and ability to perform these actions?*

This cross‐sectional survey study employed the use of a secondary data analysis to explore and understand the relationship of five older adults’ demographic characteristics with their self‐reported importance levels of seven patient engagement self‐care actions for communicating with healthcare professionals, along with their desire and ability to perform these actions. The five patient demographic characteristics selected in this study were residence in an urban or rural site, gender, age group, marital status and education level. This study used a study design approach similar to a recent study conducted by Tzeng et al. (2018), with a different self‐care focus area. The secondary data used in this study are part of a cross‐sectional survey project (Tzeng, & Pierson, 2017a). The Center for Advancing Health's Engagement Behavior Framework (CFAH, 2010, 2014a, 2014b) was used as a theoretical framework to guide the larger study that these data came from. The Engagement Behavior Framework (CFAH, 2010, 2014a, 2014b) was meant to help adults understand what they need to do to benefit from the health services available to them in the context of the US healthcare system. In other words, patient engagement is conceptualized, based on the definition used by the Center for Advancing Health (CFAH, 2010, 2014a, 2014b), as the behaviours adults need to equip to obtain the greatest benefit from the healthcare services available to them in their community.

In this study, we examined seven patient engagement behaviours required to effectively communicate with healthcare professionals, as defined in previous studies (CFAH, 2014a, 2014b; Tzeng, & Pierson, 2017a, 2017b; Tzeng, Pierson, et al., 2019). Patients’ self‐reported perception of each of the seven actions was measured using yes/no questions for each action's importance, desirability to perform and patient ability to perform. The seven self‐care actions explored in this study bordered around the following thematic areas: (a) itemizing questions and issues to discuss with a healthcare personnel before or during a healthcare appointment, (b) having a personalized list of medications and taking them to one's healthcare appointments, (c) having a basic knowledge of what one's medications are for and being willing to share with healthcare personnel, (d) being willing and open to sharing one's physical symptoms and history with healthcare personnel, (e) being willing and open to sharing one's mental symptoms and history with healthcare personnel (f) being inquisitive about the quality of care delivered by one's healthcare provider and (g) being willing and open to sharing one's concerns and ideas about their care experience (Tzeng, & Pierson, 2017a).

As for the relevance of this present study to international readers, it is commonly perceived that cultural considerations could hinder access and use of healthcare resources, due to such as communication barriers (e.g., linguistic or health literacy related), historical mistrust between patients and healthcare providers, racism and discrimination (e.g., stigma related to people with advancing age) (National Academies of Sciences, Engineering, & Medicine, 2019). These communication barriers are not unique to the United States. These barriers could happen in any community in our global village and vary from community to community (e.g., because of different cultural beliefs and local geographical characteristics). To address communication barriers, it is critical that healthcare systems and healthcare providers recognize and respect that patients’ viewpoints about health and well‐being could differ (e.g., their desire to perform certain self‐care actions to navigate through the healthcare systems) (National Academies of Sciences, Engineering, & Medicine, 2019).

### 1.2 Literature review

Two recent studies concluded that it is essential to establish a checklist of desired self‐care behaviours to involve adults in their own health and health care, such as those related to communicating effectively with healthcare professionals (Tzeng, & Pierson, 2017a, 2017b). At each patient–clinician encounter, patients often anticipate open, accommodating and unbiased medical communication from healthcare professionals, because patients want their providers to attend to their health issues (Cené et al., 2009). However, this has not been the case based on studies carried out to explore this idea. For example, in studies conducted by Sudore et al. (2009) reached the conclusion that patients perceive instructions from physicians to be ambiguous and not clearly communicated or easily understood. These findings are in agreement with many other studies (Nguyen, Hong, & Prose, 2013; Roscoe, Tullis, Reich, & McCaffrey, 2013; Scholl, Zill, Härter, & Dirmaier, 2014; Tariman, Berry, Cochrane, Doorenbos, & Schepp, 2012). A survey study conducted by Graybill et al. (2018), on both patients and healthcare professionals in the United States, reported that patients considered communication with nurses more satisfactory in understanding drug dosages, dose changes, locating departments for diagnostic investigations and how to get test results.

Siminoff, Graham, and Gordon (2006) explored the relationships between patient communicative engagements with healthcare professionals and patients’ demographic characteristics. They found that patients who were older, more educated and more affluent were more prone to participate in more communicative engagements than their younger, less educated or less affluent counterparts (Siminoff et al., 2006). A few other studies (Beverly et al., 2012, 2011; Ritholz, Beverly, Brooks, Abrahamson, & Weinger, 2014), exploring factors that hinder open communication between patients and healthcare professionals, cited that these precedents may to be due to fear of judgement, guilt or shame. These findings further emphasize the need to develop optimum and desirable approaches that would facilitate and promote active interaction between patients and their healthcare professionals. This is imperative as patients who lack the desirability and ability to effectively communicate with their healthcare professionals may limit the quality of the healthcare received and their motivation as patients to further pursue healthcare services (Hashim, 2017; Johnson, Saha, Arbelaez, Beach, & Cooper, 2004; Ozaras & Abaan, 2016; Saha, Arbelaez, & Cooper, 2003; Siminoff et al., 2006; Tzeng, Okpalauwaekwe, et al., 2019; Tzeng, & Pierson, 2017a; Zachariae et al., 2003).

## 2 MATERIALS AND METHOD

### 2.1 Study design

A secondary data analysis was conducted using the data from a cross‐sectional survey study on community‐dwelling adults living in the southern United States, 2015–2016 (Tzeng et al., 2018; Tzeng, & Pierson, 2017a). This survey study obtained ethical approvals from both the Tennessee Technological University's Institutional Review Board and the University of Saskatchewan's Ethics Committee. Participants provided written consent for their data to be used in the study. The study was carried out in accordance with the principles of the Declaration of Helsinki.

This study was conducted in a region with a total of 14 counties; one of the countries is categorized as an urban area and rest of them are rural. This region is located in the middle part of a southern state (2017 state population: 6,715,984). As for the racial composition, in 2013, 79.1% are White, and 17% are Black (Wikipedia, 2019). This region is about an hour by car via highways from the state capital. An area agency on ageing and disability was located in the urban county within this region to offer various services for residents 60 years or older and the ones with a disability. Services include, but not limited to, senior centres, home‐delivered meals, congregate meals in social settings, family caregiver support, homemaker service, personal care, adult day care, public guardianship, advocates on behalf of residents of long‐term care facilities, legal assistance, educating older adults Medicare and Medicaid beneficiaries on how to reduce and report health insurance fraud, transportation and supplemental nutrition assistance programme (Upper Cumberland Development District, 2019).

### 2.2 Conceptual framework

This study was designed based on the Engagement Behavior Framework (2010) developed by the Center for Advanced Health (CFAH). This Center's 2014 publication *Here to stay: What health care leaders say about patient engagement* drew attention to their Engagement Behavior Framework (CFAH, 2010). The framework (CFAH, 2010) was meant to evaluate the size and extent of the challenges adults face to engage in their own health and health care and what they need to do to gain from existing healthcare services (CFAH, 2010; Tzeng & Pierson, 2017a). CFAH's effort was intended to lead to purposefully pointed interventions to upkeep the ability of adults to engage in their health and health care (CFAH, 2010, 2014a, 2014b). Here, we adopted the assumption developed by previous studies (CFAH, 2010; Tzeng, & Pierson, 2017a, 2017b; Tzeng, Pierson, et al., 2019) that supporting adult patients’ desires for self‐care and ability for health system navigation could lead to reduced healthcare costs.

### 2.3 Study subjects

Convenience sampling was used to recruit community‐dwelling adult subjects 18 years and older. Participation was voluntary. A total of 250 subjects (response rate 82%) participated in the survey project. This study only includes responses from participants aged 65 years and older (*N* = 123). A detailed description of the methodology employed in the studies has been published (Tzeng et al., 2018; Tzeng & Pierson, 2017a).

To calculate the required sample size, the guideline developed by Peduzzi, Concato, Kemper, Holford, and Feinstein (1996) was used. Assuming that the proportion of positive outcomes is 0.20 (20%), the required minimum number of cases is *N* = 10 × (1/0.20) = 50 for one regression coefficient and *N* = 10 × (2/0.20) = 100 for two regression coefficients. For multiple logistic regression that would include all five demographic characteristics in the model at the same time, there would be a total of eight regression coefficients. The required minimum number of cases would *N* = 10 × 8/0.20 = 400. For this secondary data analysis and based on the sample size calculations, this study did not have a sufficient sample size for multiple logistic regression.

### 2.4 Data collection instruments

The self‐administered survey of community‐dwelling adults included two parts. The first was data collected in the *Patient Action Inventory for Self‐Care*, an 11‐category measurement inventory with a total of 57 items across 11 categories. The second part included questions to collect demographic characteristics. The full survey required about 40 min to complete. (Tzeng et al., 2018; Tzeng & Pierson, 2017a).

For the *Patient Action Inventory for Self‐Care* questionnaire, participants were asked to indicate yes or no for each action statement from three perspectives as follows: (a) Is this important to you? (b) Do you want to do this? and (c) Are you able to do this? (Tzeng et al., 2018; Tzeng & Pierson, 2017a). Participants’ answers to the seven self‐care behaviours in one of the 11 categories (Category 2: communicating with healthcare professionals, tool action items 5–11), were analysed, which were (1) making a list of questions and issues to discuss at your appointment (tool action 5); (2) listing your medications and taking your list to appointments (tool action 6); (3) being ready to talk about your medications and what they do (tool action 7); (4) sharing all your physical symptoms and history with your healthcare providers (tool action 8); (5) sharing all your mental symptoms and history with your healthcare providers (tool action 9); (6) asking questions of your provider when needed (tool action 10); (7) sharing your ideas about the care experience (tool action 11) (Tzeng & Pierson, 2017a).

The Cronbach's alpha, a measure of internal consistency or scale reliability, for the *Patient Action Inventory for Self‐Care* as a whole was 0.968 (Tzeng et al., 2018; Tzeng & Pierson, 2017a). The five demographic questions included in this study were (a) residence (1 = urban, 0 = rural); (b) gender (1 = male, 0 = female); (c) age group (1 = 65 to less than 75 years old, 2 = 75 to less than 85 years old, 3 = 85 years old or older); (d) marital status (1 = married; 2 = single, widowed and divorced; 3 = separated); and (e) education level (1 = less than high school diploma, 2 = high school diploma, 3 = associate degree, bachelor's degree or higher). Ethnicity was not included in this study due to lack of variation (Tzeng et al., 2018; Tzeng & Pierson, 2017a).

### 2.5 Data analysis

We used IBM SPSS 23.0 statistical software for analyses (IBM Corp., 2015). Statistics were calculated without adjusting for missing values; in other words, data from completed or partially completed surveys were included in the analysis. Descriptive analyses (i.e., frequency counts and percentages) were used to describe the sample. Univariate logistic regression was performed to evaluate the contribution of individual demographic characteristics. Multiple logistic regression (method = enter) was completed to evaluate the influence of the five identified demographic characteristics on the chance that older adults would report that they perceived each of included seven self‐care actions as being imperative, wanted and capable to perform by participants (yes = 1, no = 0). The alpha value was set at 0.05 for two‐tailed tests.

## 3 RESULTS

### 3.1 Demographic characteristics

Table 1 presented the descriptive statistics of the demographic characteristics and the included self‐care actions. The majority of the older adults lived in a rural area (61.8%; *N* = 76), were female (73.3%; *N* = 90), hold at least a high school diploma (66.7%; *N* = 82) and were White (90.2%; *N* = 111). Sixty (48.8%) of them were at least 65 and younger than 75 years of age. Relationship status was fairly equally divided between married (39%; *N* = 48) and single (9.9%; *N* = 49). Four (3.3%) of them did not have health insurance.

**Table 1:** Summary of the descriptive analyses of the demographic characteristics of the community‐dwelling residents aged 65 years and older and their perceptions (*N* = 123)

Demographic variables	Categories (coding for analyses)	Frequency (%)
Residential Site	Urban counties (1)	47 (38.2)
	Rural counties (0)	76 (61.8)
Gender	Female (0)	90 (73.3)
	Male (1)	23 (18.7)
	No answer (missing)	10 (8.1)
Age in years	65 to <75 years (1)	60 (48.8)
	75 to <85 years (2)	44 (35.8)
	85 years and older (3)	19 (15.4)
Marital status	Married (1)	48 (39.0)
	Single, widowed, divorced (2)	49 (39.9)
	Separated (3)	12 (9.8)
	No answer (missing)	14 (11.4)
Education	Less than high school diploma (1)	18 (14.6)
	High school diploma (2)	82 (66.7)
	Associate degree, bachelor's degree, and above (3)	23 (18.7)
Ethnic group (Choose all that apply)	White, non‐Hispanic	111 (90.2)
	White, Hispanic	6 (4.9)
	Black or African American	1 (0.8)
	American Indian or Alaska Native	5 (4.1)
	Asian	0 (0)
	Native Hawaiian, or other Pacific Islander	0 (0)
	Other race	0 (0)
Patients’ perceived importance levels	Categories (coding for analyses)	Frequency (%)
(Communication action item 1) Make a list of questions and issues to discuss at your appointment	No (0)	11 (8.9)
	Yes (1)	97 (78.9)
	No answer (missing)	15 (12.2)
(Communication action item 2) List your medications and take your list to appointments	No (0)	3 (2.4)
	Yes (1)	113 (91.9)
	No answer (missing)	7 (5.7)
(Communication action item 3) Be ready to talk about your medications and what they do	No (0)	3 (2.4)
	Yes (1)	107 (87)
	No answer (missing)	13 (10.6)
(Communication action item 4) Share all your physical symptoms and history with your healthcare providers	No (0)	2 (1.6)
	Yes (1)	114 (92.7)
	No answer (missing)	7 (5.7)
(Communication action item 5) Share all your mental symptoms and history with your healthcare providers	No (0)	7 (5.7)
	Yes (1)	104 (84.6)
	No answer (missing)	12 (9.8)
(Communication action item 6) Ask questions of your provider when needed	No (0)	1 (0.8)
	Yes (1)	117 (95.1)
	No answer (missing)	5 (4.1)
(Communication action item 7) Share your ideas about the care experience	No (0)	10 (8.1)
	Yes (1)	96 (78)
	No answer (missing)	17 (13.8)
Patients’ perceived desire levels	Categories (coding for analyses)	Frequency (%)
(Communication action item 1) Make a list of questions and issues to discuss at your appointment	No (0)	11 (8.9)
	Yes (1)	72 (58.5)
	No answer (missing)	40 (32.5)
(Communication action item 2) List your medications and take your list to appointments	No (0)	5 (4.1)
	Yes (1)	82 (66.7)
	No answer (missing)	36 (29.3)
(Communication action item 3) Be ready to talk about your medications and what they do	No (0)	5 (4.1)
	Yes (1)	82 (66.7)
	No answer (missing)	36 (29.3)
(Communication action item 4) Share all your physical symptoms and history with your healthcare providers	No (0)	6 (4.9)
	Yes (1)	81 (65.9)
	No answer (missing)	36 (29.3)
(Communication action item 5) Share all your mental symptoms and history with your healthcare providers	No (0)	11 (8.9)
	Yes (1)	72 (58.5)
	No answer (missing)	40 (32.5)
(Communication action item 6) Ask questions of your provider when needed	No (0)	3 (2.4)
	Yes (1)	87 (70.7)
	No answer (missing)	33 (26.8)
(Communication action item 7) Share your ideas about the care experience	No (0)	10 (8.1)
	Yes (1)	73 (59.3)
	No answer (missing)	40 (32.5)
Patients’ perceived ability levels	Categories (coding for analyses)	Frequency (%)
(Communication action item 1) Make a list of questions and issues to discuss at your appointment	No (0)	7 (5.7)
	Yes (1)	86 (69.9)
	No answer (missing)	30 (24.4)
(Communication action item 2) List your medications and take your list to appointments	No (0)	2 (1.6)
	Yes (1)	93 (75.6)
	No answer (missing)	28 (22.8)
(Communication action item 3) Be ready to talk about your medications and what they do	No (0)	2 (1.6)
	Yes (1)	93 (75.6)
	No answer (missing)	28 (22.8)
(Communication action item 4) Share all your physical symptoms and history with your healthcare providers	No (0)	4 (3.3)
	Yes (1)	93 (75.6)
	No answer (missing)	26 (21.1)
(Communication action item 5) Share all your mental symptoms and history with your healthcare providers	No (0)	6 (4.9)
	Yes (1)	87 (70.7)
	No answer (missing)	30 (24.4)
(Communication action item 6) Ask questions of your provider when needed	No (0)	3 (2.4)
	Yes (1)	97 (78.9)
	No answer (missing)	23 (18.7)
(Communication action item 7) Share your ideas about the care experience	No (0)	6 (4.9)
	Yes (1)	84 (68.3)
	No answer (missing)	33 (26.8)

### 3.2 Univariate logistic regression findings

Among the included patient demographic characteristics tested, univariate logistic regression analysis showed statistical significance only for marital status and the ability level to perform self‐care communication action item 7 (i.e., sharing ideas about care experience) (*p*‐value = 0.042; OR 0.069; 95% CI = 0.005–0.913) (see Table 2). This model explained between 5.4% (Cox & Snell R square)–14.3% (Nagelkerke R square) of the variance and correctly classified 93.7% of cases. Only separated marital status was statistically significant. The odds ratio of 0.069 for separated marital status (compared to married) was less than 1, indicating that the odds of being able to perform communication action item 7 is (1 − 0.069) × 100% = 93.1% lower for a separated person compared to those who were married.

**Table 2:** Univariate logistic regression predicting likelihood of reporting yes (1) to having the ability to perform the self‐care action of *sharing your ideas about the care experience* (communication action item 7) (*N* = 123)

	*B*	*SE*	Wald	*df*	*p*	Odds ratio	95% CI for odds ratio/lower	95% CI for odds ratio/upper
Marital status (Reference group: 1 = married)			4.804	2	0.091			
Marital status (2 = single)	−0.780	1.248	0.391	1	0.532	0.458	0.040	5.293
Marital status (3 = separated)	−2.667	1.314	4.117	1	0.042*	0.069	0.005	0.913

* *p* < 0.05.

### 3.3 Multiple logistic regression findings

Three multiple logistic regression models including all five demographic characteristics and with at least one statistically significant regression coefficient value are summarized in Table 3. The first model attempted to predict the chance of answering yes to the aspiration to perform self‐care communication action item 1, making a list of healthcare issues to discuss with healthcare professionals at appointments (chi‐square (8) = 12.851, *p* = 0.117, *N* = 67). Overall, this model explained between 17.5% (Cox & Snell R square)–32% (Nagelkerke R square) of the variance and correctly classified 86.6% of responses. Male gender was statistically significant. The odds ratio of 0.054 for male gender, compared to female gender, was less than 1, showing that the odds of desiring to do self‐care communication action item 1 is (1 − 0.054) × 100% = 94.6% lower for a male person compared to a female older adult.

**Table 3:** Summary of multiple logistic regression models with at least one statistically significant regression coefficient value. Three models are summarized below. (*N* = 123)

	*B*	*SE*	Wald	*df*	*p*	Odds ratio	95% CI for odds ratio/lower	95% CI for odds ratio/upper
Model 1: Predicting the likelihood of reporting yes (1) to desire to perform the self‐care action of *making a list of questions and issues to discuss at your appointment* (communication action item 1)								
Residence (1 = urban)	−1.846	1.248	2.188	1	0.139	0.158	0.014	1.822
Gender (1 = male)	−2.926	1.449	4.076	1	0.044*	0.054	0.003	0.918
Age group (reference group: 1 = 65 to <75 years)			3.926	2	0.140			
Age group: 2 = 75 and <85 years old	−2.904	1.490	3.795	1	0.051	0.055	0.003	1.018
Age group: 3 = 85 years and older	−1.914	1.753	1.192	1	0.275	0.147	0.005	4.583
Marital status (reference group: 1 = married)			0.846	2	0.655			
Marital status: 2 = single	−0.752	1.051	0.511	1	0.475	0.472	0.060	3.702
Marital status: 3 = separated	−1.171	1.412	0.687	1	0.407	0.310	0.019	4.939
Education (reference group: 1 = less than high school diploma)			3.419	2	0.181			
Education: 2 = high school	2.678	1.478	3.282	1	0.070	14.552	0.803	263.696
Education: 3 = associate degree, bachelor's degree, and above	2.707	1.822	2.209	1	0.137	14.989	0.422	532.525
Model 2: Predicting the likelihood of reporting yes (1) to desire to perform the self‐care action of *sharing your ideas about the care experience* (communication action item 7)								
Residence (1 = urban)	−1.452	1.039	1.954	1	0.162	0.234	0.031	1.794
Gender (1 = male)	−2.864	1.310	4.782	1	0.029*	0.057	0.004	0.743
Age group (reference group: 1 = 65 to <75 years)			2.505	2	0.286			
Age group: 2 = 75 and <85 years old	−1.997	1.293	2.386	1	0.122	0.136	0.011	1.711
Age group: 3 = 85 years and older	−1.061	1.621	0.428	1	0.513	0.346	0.014	8.305
Marital status (reference group: 1 = married)			2.098	2	0.350			
Marital status: 2 = single	−0.876	1.023	0.734	1	0.392	0.416	0.056	3.090
Marital status: 3 = separated	−2.042	1.419	2.072	1	0.150	0.130	0.008	2.093
Education (reference group: 1 = less than high school diploma)			2.502	2	0.286			
Education: 2 = high school	1.987	1.302	2.328	1	0.127	7.292	0.568	93.587
Education: 3 = associate degree, bachelor's degree, and above	2.197	1.674	1.723	1	0.189	8.999	0.339	239.214
Model 3: Predicting the likelihood of reporting yes (1) to be able to perform the self‐care action of *sharing your ideas about the care experience* (communication action item 7)								
Residence (1 = urban)	−1.081	1.317	0.673	1	0.412	0.339	0.026	4.487
Gender (1 = male)	−1.967	1.628	1.460	1	0.227	0.140	0.006	3.398
Age group (reference group: 1 = 65 to <75 years)			0.252	2	0.882			
Age group: 2 = 75 and <85 years old	−0.216	1.648	0.017	1	0.896	0.806	0.032	20.364
Age group: 3 = 85 years and older	−0.975	2.038	0.229	1	0.632	0.377	0.007	20.488
Marital status (reference group: 1 = married)			4.712	2	0.095			
Marital status: 2 = single	−1.254	1.644	0.581	1	0.446	0.285	0.011	7.162
Marital status: 3 = separated	−4.461	2.098	4.520	1	0.033*	0.012	0.000	0.706
Education (reference group: 1 = less than high school diploma)			2.316	2	0.314			
Education: 2 = high school	2.283	1.500	2.316	1	0.128	9.802	0.518	185.353
Education: 3 = associate degree, bachelor's degree, and above	21.573	9,511.69	0.000	1	0.998	0.001	0.000	—

* *p* < 0.05.

The second regression model evaluated the desire level to do self‐care communication action item 7 (chi‐square (8) = 11.263, *p* = 0.187, *N* = 69), which explained between 15.1% (Cox & Snell R square)–27.9% (Nagelkerke R square) of the variance and correctly classified 89.9% of responses. Male gender was statistically significant. The odds ratio of 0.057 for male gender was less than 1, demonstrating that the odds of having a desire to share their ideas about the care experience decreases by (1 − 0.057) × 100% = 94.3% for a male person compared to a female older adult.

The third model was for the level of ability to perform self‐care communication action item 7 and contained the same five demographic characteristics (chi‐square (8) = 12.394, *p* = 0.134, *N* = 75), which explained between 15.2% (Cox & Snell R square)–39.3% (Nagelkerke R square) of the variance and correctly classified 96% of responses. Separated marital status was statistically significant. The odds ratio of 0.012 for the separated marital status was less than 1, demonstrating that the odds of having the ability to do self‐care communication action item 7 decreases by (1 − 0.012) × 100% = 98.8% for a separated person compared to a person who is married.

## 4 DISCUSSION

As shown in the results, among the seven self‐care behaviours related to communicating with healthcare professionals, communication action items 1 and 7 (i.e., making a list of healthcare issues and sharing ideas about care, respectively) showed significant relationships with gender and marital status. These two findings are consistent and imply that separated older adults are not able to share their ideas about the care experience with their healthcare professionals compared with their married counterparts. In the same vein, male older adults were showed to have less desire to perform both self‐care communication action items 1 and 7 (making a list of healthcare issues and sharing ideas about care, respectively) than female older adults. This implies that male older adults had less desire to list their healthcare concerns and also less desire to share their ideas about their care experience with their healthcare professionals, compared to their female counterparts. These study's findings were not in agreement with the findings of Siminoff et al. (2006) who reported that older, more educated and more affluent patients have greater communicative engagement with physicians than their younger, less educated or less affluent counterparts. The findings of the present study could be interpreted to add to our understanding of communication disparities in variable contexts.

Furthermore, our study results showed that male older adult patients were less likely to communicate effectively with their healthcare providers, compared to their female counterparts. This finding is supported by several studies in the social science field, which asserts that females are expressive in verbal and non‐verbal communication techniques than males (Hall, 1984; Hall & Roter, 2002; Kiss, 2004; Zaharias, Piterman, & Liddell, 2004). While we acknowledge that there might be nuances, such as patient–healthcare professional gender dyads (e.g., male or female patients being more comfortable to communicate based on gender differences with the physician or nurse) that could have modified our results (Jefferson, Bloor, Birks, Hewitt, & Bland, 2013; Mast & Kadji, 2018; Shin et al., 2015). We interpret our results with caution having these in mind. Further research on these subtleties could further elucidate on how these could confound or interact with our study results.

In this study, our results emphasize the need for patient‐centred care in health practice. Healthcare professionals (i.e., nurses, nursing practitioners and other professional healthcare providers) should be prepared to support older adults, regardless of their gender or other demographic characteristics, to find their voice in holding effective dialogue with their healthcare professionals. This is because professional nurses are entrusted with the responsibility to provide holistic care to patients as they deal with them more closely (Ozaras & Abaan, 2016). Considering the overwhelming and inundating nature of the work of nurses, it is vital that nurses and other healthcare providers develop and apply pragmatic measures to foster a conducive environment where patients can feel free to communicate or share their concerns about their care. For example, healthcare providers may introduce the *QuestionBuilder App* (Agency for Healthcare Research & Quality, 2019)—a smartphone‐friendly application developed by US Agency for Healthcare Research and Quality—to older adults to help them prepare and organize their questions for medical encounters. Nurses could provide tools, such as the Iconic Pain Assessment Tool (Lalloo & Henry, 2011), to assist older adults self‐reporting of pain quality, intensity and location. Nurses could also encourage older adults to document their issues or concerns prior to arrival or during waiting time at the clinic. Healthcare professionals need to be mindful that, compared to female older adults, male older adults have a tendency to shy away from active involvement in their own care.

Thus, the intent of patient education could be to support male older adults in making a list of health‐related questions or in sharing their ideas about their healthcare needs by, for example, providing a paper‐based or internet‐based self‐report pain assessment tool. In addition, healthcare professionals should assess separated older adults’ ability to share their ideas about their care experience and, more specifically, the facilitators and barriers that affect their comfort levels in communicating with their healthcare providers, including challenges in accessing an internet patient communication portal. Based on these findings, older adults and healthcare professionals may jointly develop an education goal to assist separated older adults in performing the self‐care behaviour of sharing their ideas about the care experience. This is essential as sometimes the goals of the older adults and the healthcare professionals are completely incongruent, and only effective communication can successfully resolve this situation.

On a global scene, communication barriers could happen in any community and vary across communities because of different cultural beliefs and local geographical characteristics. To address communication barriers, it is important that healthcare providers first seek to understand each older adult's unique perspectives about health and well‐being. The findings in this study could serve as a reference to help older adults build up their self‐care capacity when communicating with healthcare professionals (National Academies of Sciences, Engineering, & Medicine, 2019). Also, it is essential for nurses and other healthcare professionals working in both inpatient and outpatient settings to be familiar with the services provided at the local area agency on ageing and disability (or a similar government‐funded agency) to support older adults’ needs in self‐care behaviours. Referring older adults to the free or low‐cost services or programmes provided by an area agency on ageing and disability with a goal to improve older adults’ confidence or provide support in communicating with healthcare professionals could be beneficial.

### 4.1 Study limitations and future directions

A significant limitation of this project was the relatively low sample size which may have underpowered the results of the analyses. The data being concentrated to the southern part of the United States may not be appropriately representative (owing to the sample size) or generalizable to other regions in the United States. Future research could also investigate the associations between older adults’ perceptions of self‐care behaviours related to communicating with their healthcare professionals and their race/ethnicity, religious beliefs and/or preferred language. This may be a promising avenue for research following the findings set by Siminoff et al. (2006), who found that White patients were reported to have more expressive communication with their healthcare providers than their non‐White counterparts, or Cené et al. (2009), who found that significant differences in quality patient–physician communication between Black and non‐Black patients (e.g., Blacks had shorter physician visit and less communication than Whites).

## 5 CONCLUSIONS

This study concluded that among the seven self‐care actions for communicating with healthcare professionals, two self‐care actions—*making a list of questions and issues to discuss at your appointment* (communication action item 1) and *sharing your ideas about the care experience* (communication action item 7)—were associated with gender and marital status among community‐dwelling older adults. Healthcare professionals should intentionally observe and assess patients who may be separated or male older adults in their ability to communicate effectively and expressly with their healthcare providers, especially with older adults who are more susceptible to visit the emergency rooms or clinic. This has been supported by our study to be an essential requirement for patient‐centred care and patient engagement among older adults.

## CONFLICT OF INTEREST

No conflict of interest has been declared by the authors.

## ETHICAL APPROVAL

This study obtained ethical approvals from both the Tennessee Technological University's Institutional Review Board and the University of Saskatchewan's Ethics Committee. Participants provided written consent for their data to be used in the study. The study was carried out in accordance with the principles of the Declaration of Helsinki.
